# Supplementary material for: Self assembly of HIV-1 Gag protein on lipid membranes generates PI(4,5)P2/Cholesterol nanoclusters
Source: Sci Rep. 2016 Dec 23;6:39332. doi: 10.1038/srep39332 (PMC5180241; doi:10.1038/srep39332)
Supplement: Supplementary Information [file srep39332-s1.pdf]

Self assembly of HIV-1 Gag protein on lipid membranes generates $PI(4,5)P_2$/Cholesterol nanoclusters.

Naresh Yandrapalli[1], Quentin Lubart[2], Hanumant S. Tanwar[3], Catherine Picart[2], Johnson Mak[3], Delphine Muriaux[1,*], and Cyril Favard[1,*]

[1]CNRS-Université Montpellier, Centre d'études d'agents Pathogenes et de Biotechnologies pour la Santé, Montpellier, France
[2]CNRS-Université Grenoble, Institut National de Physique, Grenoble, France
[3]Deakin University, School of Medicine, Melbourne, Australia
[*]delphine.muriaux@cpbs.cnrs.fr, cyril.favard@cpbs.cnrs.fr

ABSTRACT

Supplemental Information

Effect of probe and probe concentration on the fluorescence quenching

We first conducted experiments on the same LUVs as the one described in[1] with the dye originally used (BT-PIP_2) and compared to the results obtained using TF-PIP_2. Figure 1A shows a good reproduction of the BT-PIP_2 quenching published in[1]. It also shows that substituting BT-PIP_2 by TF-PIP_2 does not change the effect observed after addition of MARCKS peptide, this either at 1% or 2% molar concentration. We also tested the quenching concentration dependency of TF-PIP_2 upon addition of different FL-Gag concentrations (Fig.1B). Again, we did not observe any drastic change with different dye molar ratios. All these results show a weak dependency of the induced PIP_2 clustering upon the nature of the fluorescent dye and its molar concentration in the LUVs. We then tested these effects on SLBs basic membranes. Figure 1 C and D shows the change in fluorescence of either TF- or BT-labeled PIP_2 after FL-Gag addition at different concentrations. At low FL-Gag concentration (i.e. equivalent to small FL-Gag/PIP_2^{acc}), we could see differences depending on the molar concentration and the nature of the fluorophore in the normalized fluorescence value (0.4 for TF-PIP_2 and 0.2 for BT-PIP_2). Importantly, these differences faded with increasing FL-Gag concentration. At FL-Gag concentrations above 200 nM, the fluorescence of either different dye molar ratio (Fig. 1 D) or chemical nature (see figures 4 and 5 of the main manuscript) always fall down to 0.2 ± 0.1. We concluded that, at labeled lipid saturating FL-Gag concentrations, the nature and the concentration of the dye does not have any influence on the relative fluorescence decrease.

Quenching/Unquenching and membrane curvature

BT-labeled lipids clustering generally induce fluorescence quenching on LUVs (fig. 1A), i.e. fluorescence decrease[1,2]. Therefore, unquenching observed in this study upon Gag and its mutant addition to TF-PIP_2 containing LUVs was unexpected. Nevertheless, this TF-PIP_2 unquenching has already been described with Newcastle disease virus M protein addition on LUVs[3]. Shnyrova *et al.* suggested the decrease of dynamic quenching due to lipid clustering upon addition of virus M protein to be responsible for this unquenching[3].

We examined a possible role for the dynamic quenching to produce opposite effects on LUVs and SLBs. Two different types of fluorescence quenching may occur, i.e. the dynamic quenching and the static quenching. Overall, the total quenching can be simply described by :

$$Q_T = Q_d + Q_s \tag{1}$$

To simplify, dynamic quenching reflect the possibility of the quencher to collide with an excited fluorescent molecule. It is proportional to the quencher concentration (here TF-PIP_2) and the temperature. Static quenching reflects the possibility of the quencher to form a complex with the quenched molecule at the ground state. This complex then becomes non fluorescent. Both type of quenching (dynamic and static) induce a fluorescence intensity decrease with increasing concentration of the quencher. But, only an increasing dynamic quenching will result in decreasing the fluorescence lifetime of the quenched molecule.

Therefore to understand the respective role of dynamic and static quenching of the TF-PIP_2 after FL-Gag self assembly on SLBs and LUVs, we acquired fluorescence lifetime images. Fluorescence lifetime of a dye exposed to dynamic quenchers is expected to decrease with increasing quencher concentration. Figure 2 part A to D, illustrate the change in lifetime observed on LUV (part A and C) or SLB (part B and D) upon FL-Gag addition. Interestingly an opposite tendency has been observed. In the case of LUV, before addition of FL-Gag, the lifetime distribution is centered at $\tau_1 = 3.2 \pm 0.1$ ns and it is moved to a higher lifetime value $\tau_2 = 5.1 \pm 0.8$ ns after FL-Gag addition (FL-Gag/TF-PIP_2^{acc}=10). On the opposite, SLB already exhibit two different lifetimes respectively centered at $\tau_1 = 3.6 \pm 0.3$ and $\tau_1 = 4.4 \pm 0.4$ before FL-Gag addition. The first lifetime centered at 3.6 ± 0.3 is close to the one observed for LUVs and is likely due to the presence of vesicles not totally fused with the rest of the SLB. Upon FL addition, the mean lifetime decreases slightly in the case of SLB instead of increasing. The photophysics of TF lipid derivatives is poorly documented[4]. Nevertheless, Karolin *et al.* measured lifetimes of tetra-methyl bodipy (3,3',4,4'-difluoro-1,3,5,7-tetramethyl-4-borato-3a-azonia-4a-aza-s-indacene) which is the closest chemical structure to the TF lipid derivatives. They showed the existence of two different lifetimes $\tau_1 = 5.3$ ns in acetone and $3.0 \leq \tau_2 \leq 3.7$ ns when the dye was quenched with different dynamic quenchers[5]. Our fluorescence lifetime are consistent with these values and suggest that before FL-Gag addition, the dynamic self-quenching contribution to the total self-quenching is higher in curved membranes (LUVs vs SLBs) than after FL-Gag addition.

As represented in the scheme of figure 2E, multimerizing Gag and its mutants can impose a strong local curvature to the membranes. This curvature is opposite to the one of LUVs and could have therefore a tendency to induce local "flatening" of LUVs, whereas, on the opposite it will induce a local curvature on flat SLBs.

Formulated in terms of respective quenching type contributions, it can be written :

$$Q_{Gag} = Q'_d + Q'_s \qquad \text{with} \begin{cases} Q'_d << Q_d, & \text{for LUV} \\ Q'_d > Q_d, & \text{for SLB} \end{cases} \qquad \text{while} Q'_s > Q_s \text{for LUV and SLB} \tag{2}$$

The two components of quenching are then additive in the case of SLB and opposite in the case of LUV. This explains why the fluorescence changes seen with TF-PIP_2 are inverted in infinite flat membranes (SLB) and curved membranes (100nm LUVs) upon Gag addition and why their absolute value is more important in SLB than in LUV. Supporting this hypothesis, MARCKS peptide, which is not inducing any curvature, is leading to the same overall quenching of TF-PIP_2 on flat and curved membranes (see fig.3 A and B of the main text).

Forster Energy Transfer between TF and BT labeled lipids

TF-Chol and BT-PIP_2 have been recently shown to exhibit Forster resonance energy transfer (FRET)[6]. We examined the possibility for TF-SPM (donor) and BT-PIP_2 (acceptor) to achieve FRET in the "basic" SLB used in Fig.3 of the main text (lipid composition is given in TableS1). A typical x,y image of a basic SLB labeled with TF-SPM (green) and BT-PIP_2(red) shows the good mixing properties of both dyes (fig.3A). To test the occurence of FRET in these SLBs, we repeated photobleaching of the acceptor (BT-PIP_2) and monitored the fluorescence changes of both TF-SPM and BT-PIP_2 (n=20 on 4 different SLBs). As depicted in fig.3B BT-PIP_2 exhibit a fluorescence recovery after bleaching with a fractional recovery close to 1 (as in fig. 2 of the main text). Interestingly, immediately after the end of the acceptor photobleaching pulse, TF-SPM exhibit an apparent increase in fluorescence that is a characteristic of a loss in FRET. Moreover, this increased TF-SPM fluorescence decreases with increasing fluorescence of BT-PIP_2 showing that FRET increase again with unbleached lipid remixing. This clearly confirm the existence of FRET between TF-SPM and BT-PIP_2 when these two lipid derivatives are present in the SLB.

Donor FRET efficiency is a function of acceptor concentration in the lipid bilayer[7]. FRET decreases when acceptor concentration decrease, leading to donor fluorescence to increase. This is what we observed in fig.3C, and, more interestingly, this is also what is seen in fig. 4C of the main text. This shows that during PIP_2 clustering the outside cluster BT-PIP_2 (acceptor) concentration is decreasing whereas the outside cluster TF-SPM (donor) concentration is unchanged (fig.3C). This can be interpreted as a nano-unmixing of SPM and PIP_2 during FL-Gag multimerization (see fig. 4E of the main text).

Detailed material and methods

Reagents

Egg phosphocholine (EPC), 1,2-oleoyl-sn-glycero-3-phosphocholine (DOPC), Brain L-α-phosphatidylserine (BPS), 1,2-oleoyl-sn-glycero-3-phosphoserine (DOPS), Liver phosphoethanolamine (LPE), Cholesterol, Brain Sphingomyelin (BSPM), Ovine Brain Ganglioside GM1 (GM1), Brain Phosphatidylinositol 4,5-biphosphate (BPI(4,5)P_2) Topfluor® PI(4,5)P_2 (TF-PI(4,5)P_2), Topfluor® Sphingomyelin (TF-SPM) and Topfluor® cholesterol (TF-Chol) were purchased from Avanti Polar lipids. C16-Bodipy® TMR Phosphatidylinositol 4,5-bisphosphate was purchased from Echelon Inc. Alexafluor® 594 C5-malemide probe

for labelling protein, Cholera Toxin Subunit B Alexafluor® 647 conjugate for labelling GM1 and 1,1'-Dioctadecyl-3,3,3',3'-Tetramethylindocarbocyanine Perchlorate (DiIC18) as the control lipid analogue were purchased from ThermoFisher Scientific, Inc. Tris(2-carboxyethyl)phosphine hydrochloride (TCEP) and Poly-vinyl alcohol, Mowiol®28-99 were purchased from Sigma-Aldrich.

Model membranes

Large Unilamellar Vesicles (LUVs)

Liposomes of specific compositions were prepared using a standard protocol. A lipid thin film obtained by high-pressure vacuum evaporation of solvents was hydrated with our working buffer (Hepes-10mM, KCl-150mM, and 2mM EDTA, pH 7.4). After freeze-thawing (10 cycles) and bath sonication for 20 min, 100nm diameter liposomes were produced by extrusion using Avanti® Mini-Extruder. Liposomes were used within 24h and concentration of the liposomes was controlled using phospholipid assay kit (Sigma-Aldrich).

Supported Lipid Bilayers (SLBs)

In order to make SLBs, microscope coverslips were pre-treated by bath sonicating in 5% SDS solution for 1 h and incubated for 20 min in freshly made piranha (3:1 vol:vol H_2SO_4:H_2O_2) solution, then thoroughly washed with milliQ water and prevented from drying. 30 nm diameter liposomes were prepared at 1mg.mL^{-1} concentration in a citrate buffer (NaCitrate 10mM, 100mM NaCl, and 0.5 mM EGTA, pH 4.6) by successive extrusion steps, diluted to 0.1mg.mL^{-1} and spread over the pre-treated coverslips. To produce defect free SLBs, the vesicle fusion was allowed to take place for 45 min at 37°C. The SLBs were then thoroughly washed with citrate Buffer. Just before experiments, they were stabilized with working buffer.

Giant Unilamellar Vesicles (GUVs)

GUVs were produced using gel assisted formation reported by Weinberger et al.[8], with some minor modifications. Specific composition dried lipid films (1 mg.ml^{-1}) layered on PVA coated cover slips were hydrated using sucrose containing buffer and incubated overnight at varied temperatures depending on the lipid composition. GUVs thus produced were used within three days. Care was taken to maintain the equi-osmolality of the solutions throughout the experiments.

Proteins expression and purification

Recombinant Gag FL, WM and P39 were expressed in E. coli and purified using affinity and size exclusion chromatography[9]. Purity of all protein preparations were confirmed by SDS-PAGE, mass spectrometry and western blot analysis using an anti-HIV Gag specific monoclonal antibody. Matrix protein (MA) and PH-EFA6 were purified in-house using a standard protocol. Prokaryotic expression of both the proteins was done by transfecting the vectors into BL-21 E.coli cells. A 500 mL culture was allowed to grow and IPTG induction of protein expression was done for 4h at 37°C. On expression, bacteria was pelleted and lysed using a wash/lysis buffer (50mM Sodium Phosphate, 250mM NaCl, 10mM imidazole and 0.02% β -mercaptoethanol with pH 7.0), followed by sonication for 2 min in the presence of protease inhibitors and DNAase. The cell-lysate was centrifuged at 20,000 g for 15 min at 4°C, to separate the debris from the expressed protein. Supernatant containing his-tagged protein was added with Ni-NTA agarose beads and left at least for one hour at 4°C on a rocker. After incubation, the supernatant was passed through propylene column and washed with wash/lysis buffer. To free the protein bound to Ni-NTA agarose beads, they were incubated in elution buffer (wash/lysis buffer + 250mM imidazole, pH 7.0) for 1h at 4°C on a rocker. Finally, PD-10 column was used to change the buffer to 100mM Tris, 0.5M NaCl, 0.02% β-mercaptoethanol, pH 7.4. Purified proteins were analyzed using SDS-PAGE and stored at -80°C, until use. Protein labelling was done for Gag with Alexafluor® 594 C5-maleimide according to manufacturer's guidelines.

Fluorescence Lifetime Imaging

Fluorescence lifetime images were acquired with the Zeiss LSM 780 confocal microscope equipped at a non descanned output with a high speed hybrid detector (HPM-100-40) and SPC-830 time-correlated single-photon counting (TCSPC) electronics (Becker & Hickl). A 40x, NA 1.3 objective was used to image the sample. Excitation wavelength of a Chameleon Ultra-II (Coherent Inc.) was tuned to 930 nm and used at low intensity to avoid photobleaching. The acquisition time was variable ($5 < t < 20$ min) in order to keep the number of photons above 3000 for each decay in each pixel of the image. This authorize to keep the fluorescence lifetime measurement accuracy constant ($< 2\%$) in the presence or absence of quenching and strongly decrease the contribution of background. The number of photons acquired per second was kept under the pile-up threshold (10^6 cts.s^{-1}). Fluorescence decays were fitted with a single exponential and a generated instrument response function (IRF) using the SPCImage software of Becker & Hickl.

References

1. Gambhir, A. *et al.* Electrostatic sequestration of PIP_2 on phospholipid membranes by basic/aromatic regions of proteins. *Biophys J* **86**, 2188–2207; DOI:10.1016/S0006-3495(04)74278-2 (2004).
2. Sarmento, M. J., Coutinho, A., Fedorov, A., Prieto, M. & Fernandes, F. Ca^{2+} induces PI(4,5)P_2 clusters on lipid bilayers at physiological PI(4,5)P_2 and Ca^{2+} concentrations. *Biochim Biophys Acta* **1838**, 822–830; DOI:10.1016/j.bbamem.2013.11.020 (2014).
3. Shnyrova, A. V. *et al.* Vesicle formation by self-assembly of membrane-bound matrix proteins into a fluidlike budding domain. *J Cell Biol* **179**, 627–633; DOI:10.1083/jcb.200705062 (2007).
4. Boldyrev, I. A. *et al.* New Bodipy lipid probes for fluorescence studies of membranes. *J Lipid Res* **48**, 1518–1532; DOI:10.1194/jlr.M600459-JLR200 (2007).
5. Karolin, J., Johansson, L. B.-A., Strandberg, L. & Ny, T. Fluorescence and absorption spectroscopic properties of dipyrrometheneboron difluoride (Bodipy) derivatives in liquids, lipid membranes, and proteins. *J Am Chem Soc* **116**, 7801–7806; DOI:10.1021/ja00096a042 (1994).
6. Wang, Y.-H., Bucki, R. & Janmey, P. A. Cholesterol-dependent phase-demixing in lipid bilayers as a switch for the activity of the phosphoinositide-binding cytoskeletal protein gelsolin. *Biochemistry*; **55**, 3361–3369 DOI:10.1021/acs.biochem.5b01363 (2016).
7. Fung, B. K. & Stryer, L. Surface density determination in membranes by fluorescence energy transfer. *Biochemistry* **17**, 5241–5248; (1978).
8. Weinberger, A. *et al.* Gel-assisted formation of giant unilamellar vesicles. *Biophys J* **105**, 154–164; DOI:10.1016/j.bpj.2013.05.024 (2013).
9. McKinstry, W. J. *et al.* Expression and purification of soluble recombinant full length hiv-1 pr55(gag) protein in escherichia coli. *Protein Expr Purif* **100**, 10–18; DOI:10.1016/j.pep.2014.04.013 (2014).
10. Ingólfsson, H. I. *et al.* Lipid organization of the plasma membrane. *J Am Chem Soc* **136**, 14554–14559; DOI:10.1021/ja507832e (2014).
11. Keller, H., Kräusslich, H.-G. & Schwille, P. Multimerizable HIV Gag derivative binds to the liquid-disordered phase in model membranes. *Cell Microbiol* **15**, 237–247; DOI:10.1111/cmi.12064 (2013).

c

Tables and Figures

Table 1. Different SLB lipid composition used for the dual labelled lipid experiments

TF-SPM Labeled						TF-Chol Labeled					
Substituted Basic		Inner Leaflet ♮		Rafts♯		Substituted Basic		Inner Leaflet ♮		Rafts♯	
Egg PC	66	Egg PC	17	DOPC	31	Egg PC	66	Egg PC	17	DOPC	31
Brain PS	30	Brain PS	17	DOPS	6	Brain PS	30	Brain PS	17	DOPS	6
Brain PI(4,5)P_2	1	Brain PI(4,5)P_2	1	Brain PI(4,5)P_2	6	Brain PI(4,5)P_2	1	Brain PI(4,5)P_2	1	Brain PI(4,5)P_2	6
		Brain SM	8	Brain SM	29			Brain SM	9	Brain SM	30
		Cholesterol	30	Cholesterol	25			Cholesterol	29	Cholesterol	24
		Liver PE	25	GM1	1			Liver PE	25	GM1	1
BT-PIP_2	1	BT-PIP_2	1	BT-PIP_2	1	BT-PIP_2	1	BT-PIP_2	1	BT-PIP_2	1
TF-SPM	1	TF-SPM	1	TF-SPM	1	TF-Chol	1	TF-Chol	1	TF-Chol	1

♮ Adapted from[10].
♯ Adapted from[11]

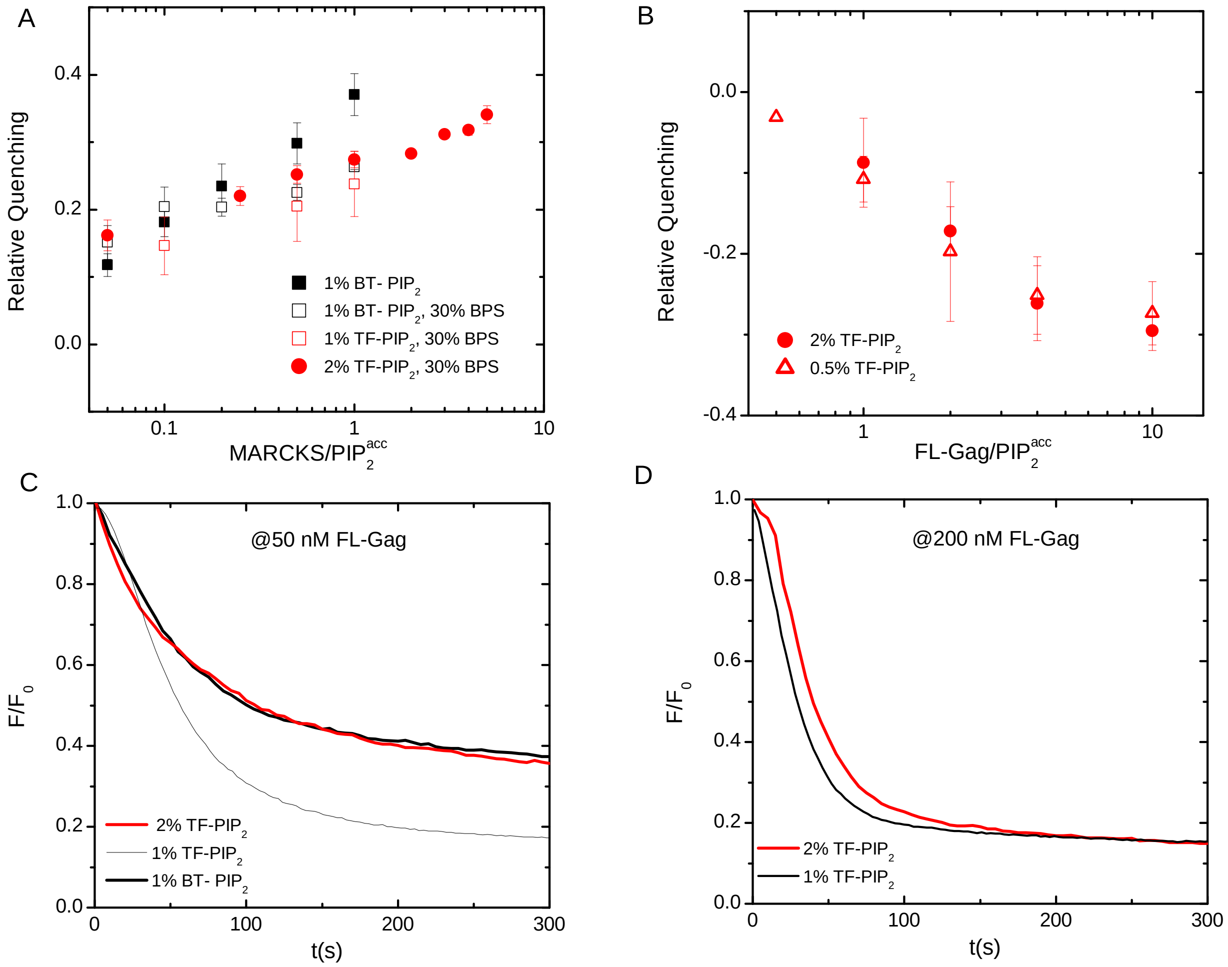

Figure 1. Quenching and unquenching observed for different Bodipy derivatives labeled lipids at different molecular ratio in LUVs and SLBs Part A : Effect of Marcks peptide on fluorescence quenching of Bodipy derivatives labeled lipids in LUVs. Black : Relative quenching of BT-PIP$_2$ in different LUVs (Closed square, 98% PC, 1% PIP2, Open square : basic composition. Red : Relative quenching of TF-PIP$_2$ at 1% mol (open square) or 2% mol(closed circle) in basic LUVs. Part B : Effect of two different TF-PIP$_2$ molar concentrations (2% and 0.5%) on the fluorescence unquenching induced by FL-Gag in basic LUVs. Part C,D : Typical fluorescence change after addition of 50nM (Part C) or 200nM (Part D) FL-Gag of different Bodipy derivatives labeled PIP$_2$ at different molar concentrations (1% or 2%) on basic SLBs.

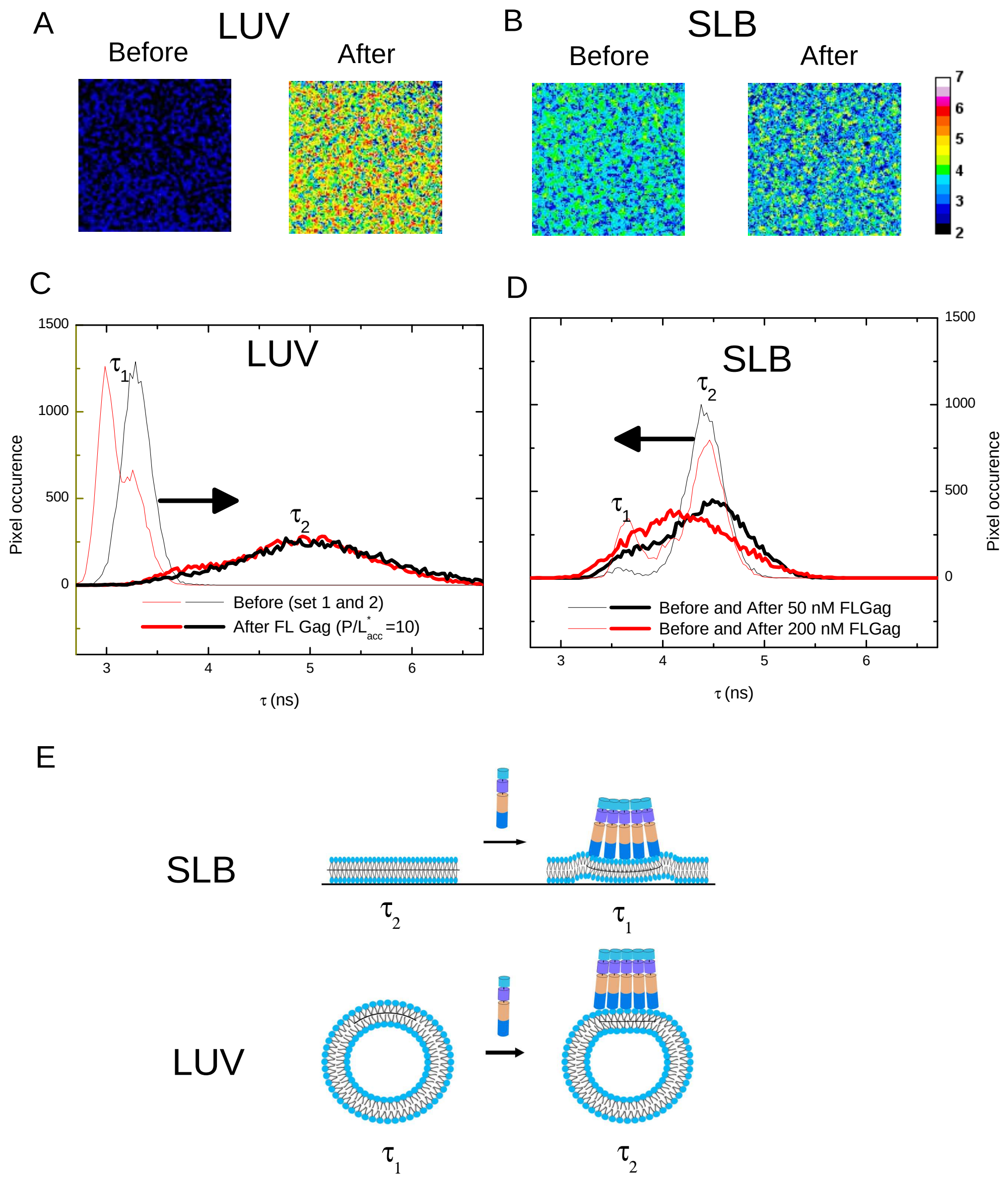

Figure 2. **TF-PIP$_2$ fluorescence lifetime change upon FL-Gag addition** Part A and B : Fluorescence lifetime images before and after addition of FL-Gag in a LUV solution (part A, P/PIP$_2^{acc}$=10) or on SLB (part B, 200nM). Part C and D : Fluorescence lifetime distributions before and after addition of FL-Gag. Part C exhibit two different measurements (red and black) in two different LUV solutions before (thin line) and after (thick line) addition of FL-Gag at P/PIP$_2^{acc}$=10 ratio. Black arrows shows the direction of the change on the mean lifetime from short (τ_1) to long (τ_2). Part D exhibit two different measurements on two different SLBs before (thin line) and after (thick line) addition of FL-Gag at 50nM (black) or 200 nM (red). Black arrows shows the direction of the change on the mean lifetime from long (τ_2) to short (τ_1). Part E : Schematic explanation of the opposite change in fluorescence lifetimes upon addition of FL-Gag for LUV and SLB containing TF-PIP$_2$. See text for detailed explanation.

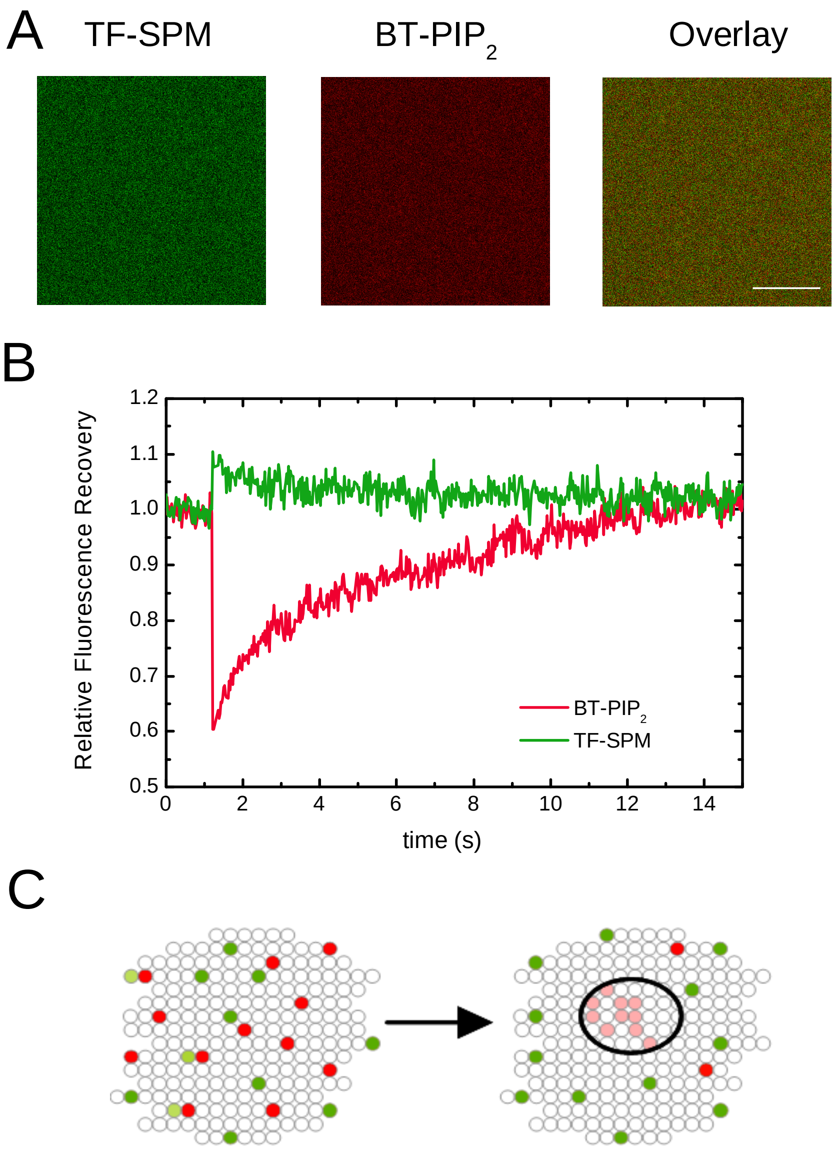

Figure 3. **SLB TF-SPM fluorescence increase upon BT-PIP$_2$ photobleaching** Part A: Confocal Laser Scanning images of basic SLB (part A) labeled with TF-SPM (green), BT-PIP$_2$ (red) and their overlay showing a good miscibility at a submicrometer scale. Part B : Mean recovery curve (n= 20 on 4 different SLBs) obtained after pulsed photobleaching of BT-PIP$_2$. Part C : Schematic representation of unmixing due to FL-Gag multimerization. Concentration of BT-PIP$_2$ out of the clusters is decreasing, inducing an increase in fluorescence of TF-SPM
